# Impact of *APOE* ε4 Carrier Status on Associations Between Subthreshold, Positive Amyloid-β Deposition, Brain Function, and Cognitive Performance in Cognitively Normal Older Adults: A Prospective Study

**DOI:** 10.3389/fnagi.2022.871323

**Published:** 2022-05-23

**Authors:** Dong Woo Kang, Sheng-Min Wang, Yoo Hyun Um, Nak-Young Kim, Chang Uk Lee, Hyun Kook Lim

**Affiliations:** ^1^Department of Psychiatry, Seoul St. Mary's Hospital, College of Medicine, The Catholic University of Korea, Seoul, South Korea; ^2^Department of Psychiatry, Yeouido St. Mary's Hospital, College of Medicine, The Catholic University of Korea, Seoul, South Korea; ^3^Department of Psychiatry, St. Vincent's Hospital, College of Medicine, The Catholic University of Korea, Seoul, South Korea; ^4^Department of Psychiatry, Keyo Hospital, Uiwang, South Korea

**Keywords:** *APOE* ε4 allele, subthreshold amyloid-beta, regional homogeneity, functional connectivity, cognitively normal older adults

## Abstract

**Background:**

A growing body of evidence suggests a deteriorating effect of subthreshold amyloid-beta (Aβ) accumulation on cognition before the onset of clinical symptoms of Alzheimer's disease (AD). Despite the association between the Aβ-dependent pathway and the *APOE* ε4 allele, the impact of this allele on the progression from the subthreshold Aβ deposits to cognitive function impairment is unclear. Furthermore, the comparative analysis of positive Aβ accumulation in the preclinical phase is lacking.

**Objective:**

This study aimed to explore the differential effect of the *APOE* ε4 carrier status on the association between Aβ deposition, resting-state brain function, and cognitive performance in cognitively normal (CN) older adults, depending on the Aβ burden status.

**Methods:**

One hundred and eighty-two older CN adults underwent resting-state functional magnetic resonance imaging, [^18^F] flutemetamol (FMM) positron emission tomography, a neuropsychological battery, and *APOE* genotyping. We evaluated the resting-state brain function by measuring the local and remote functional connectivity (FC) and measured the remote FC in the default-mode network (DMN), central-executive network (CEN), and salience network (SN). In addition, the subjects were dichotomized into those with subthreshold and positive Aβ deposits using a neocortical standardized uptake value ratio with the cut-off value of 0.62, which was calculated with respect to the pons.

**Results:**

The present result showed that *APOE* ε4 carrier status moderated the relationship between Aβ deposition, local and remote resting-state brain function, and cognitive performance in each CN subthreshold and positive Aβ group. We observed the following: (i) the *APOE* ε4 carrier status-Aβ deposition and *APOE* ε4 carrier status-local FC interaction for the executive and memory function; (ii) the *APOE* ε4 carrier status-regional Aβ accumulation interaction for the local FC; and (iv) the *APOE* ε4 carrier status-local FC interaction for the remote inter-network FC between the DMN and CEN, contributing higher cognitive performance in the *APOE* ε4 carrier with higher inter-network FC. Finally, these results were modulated according to Aβ positivity.

**Conclusion:**

This study is the first attempt to thoroughly examine the influence of the *APOE* ε4 carrier status from the subthreshold to positive Aβ accumulation during the preclinical phase.

## Introduction

Amyloid-β (Aβ) accumulation differentiates Alzheimer's disease (AD) from other neurodegenerative diseases (Jack et al., 2018). Additionally, the Aβ deposition has been known to progress non-linearly in decades before the onset of clinical symptoms of AD (Jack et al., 2013). In this regard, the preclinical stage of AD has therefore been defined as the presence of Aβ pathology without signs of significant cognitive impairment due to AD dementia (Knopman et al., 2012). In addition, the period before AD symptoms, which has become apparent, also attracts clinical attention as the right time for primary intervention because the Aβ-dependent pathophysiology is not yet fully advanced. Although this period could be overlooked in the clinical field due to the lack of apparent clinical symptoms, preclinical AD participants have demonstrated an increased risk of transition to MCI and AD (Knopman et al., 2012), as well as the deteriorating effects on the cognitive performance, brain structure, and function (Hedden et al., 2009; Mattsson et al., 2014; Baker et al., 2017).

Amyloid-β deposition is evaluated using Aβ-positron emission tomography (PET) for the early detection of preclinical AD and there has been renewed interest in Aβ accumulation below the threshold for a positive scan (Bischof and Jacobs, 2019). Even among individuals with a negative PET scan, 65% showed an early autopsy stage of Aβ accumulation (Thal phase 2), and 15% displayed an advanced stage (Thal phase 4 or 5) (Salloway et al., 2017). Additionally, specific brain regions, including the orbitofrontal cortex, the anterior cingulate cortex, and the precuneus, are prone to the earliest Aβ accumulation compared with other brain regions (Sojkova et al., 2011; Driscoll et al., 2012; Villeneuve et al., 2015). In addition, the regional Aβ deposits predicted cognitive decline more accurately than global deposition in cognitively normal (CN) older adults with subthreshold Aβ accumulation (Farrell et al., 2018). Moreover, the subthreshold Aβ deposits were associated with functional impairment (Insel et al., 2017), brain atrophy (Mattsson et al., 2014), and dysfunction of the brain functional networks (Palmqvist et al., 2017), and predicted further Aβ and tau deposition (Leal et al., 2018; Farrell et al., 2021).

*APOE* ε4 allele has been demonstrated to modulate the penetrance and weight of Aβ pathophysiological cascade (Frisoni et al., 2022) and to account for the largest proportion of the genetic risk factors for sporadic AD (Sims et al., 2020). In this regard, the *APOE* ε4 allele has been known to increase the risk of sporadic AD occurrence in a dose-dependent manner (Corder et al., 1993). The *APOE* ε4 allele has also been reported to increase Aβ production (DeMattos et al., 2004), reduce the clearance of Aβ (Castellano et al., 2011), and affect tau binding (Small et al., 2009). Lastly, *APOE* ε4 fragments have been demonstrated to interact synergistically with AD pathology, deteriorating the degree of neurodegeneration (Andrews-Zwilling et al., 2010; Bien-Ly et al., 2011). Therefore, the progression of sporadic AD cannot be fully understood without the consideration of the *APOE* genotype.

In addition, the changes in brain function have been demonstrated to precede those in the brain structure and track pathophysiological processes in the preclinical phase (Jack et al., 2013). Among the various methodologies employed for evaluating brain function, functional connectivity (FC) is a widely used and reliable method for evaluating functional interactions in the brain connectome (Biswal et al., 2010). In addition, the earliest accumulation of Aβ is known to affect FC (Palmqvist et al., 2017), and existing research has recognized the impact of the *APOE* ε4 allele on brain function and cognitive performance in the preclinical phase of AD. Previous research has found disrupted FC from the precuneus in the *APOE* ε4 carrier of the CN without positive Aβ deposition (Sheline et al., 2010). In another study, the authors demonstrated an altered within-network FC in the *APOE* ε4 carrier of the CN (Wu et al., 2016). Recently, investigators have reported a simultaneous structural and functional disruption that mediates memory impairment in the CN with the *APOE* ε4 allele (Li et al., 2021). However, the generalizability of this previous research is limited because most of these studies lacked information on Aβ deposition; they only evaluated the remote FC of predefined brain regions and showed results with weak statistical significance.

While a static FC is based on the assumed temporal stationarity of functional networks, the dynamic FC considers the dynamic nature of brain activity in faster timescales by selecting a time window that is shifted in time by a fixed number of data points (Hutchison et al., 2013). This dynamic FC has shown a significant association with the subthreshold Aβ accumulation in the preclinical phase; however, the interaction with the *APOE* ε4 allele was not evaluated in this prior study (Hahn et al., 2019). Moreover, results from dynamic FC must be interpreted with caution due to the arbitrariness of dynamic FC parameters and its vulnerability to physiological noise that could drive dynamic states (Laumann et al., 2017). These factors contribute to weaker reliability and reproducibility of dynamic FC than a static FC (Abrol et al., 2017).

Together, these studies provide important insights into the subthreshold Aβ, the pivotal role of the *APOE* ε4 allele, and the FC biomarker in the trajectory of AD. However, there remains a paucity of comprehensive evidence on the impact of *APOE* genotype on associations between subthreshold, positive Aβ accumulation, brain function, and cognitive performance in the preclinical phase of AD.

In this regard, the current study classified the CN group according to the presence of the *APOE* ε4 allele in each CN group with subthreshold Aβ deposition (CN sub-Aβ) and those with positive deposition (CN Aβ+). We aimed to evaluate the differential impact of the *APOE* ε4 carrier status on the association between the subthreshold, positive Aβ accumulation, FC, and cognitive performance in the preclinical phase. Additionally, local and remote FC evaluation of functional synchronization at different spatial scales in the brain connectome (Sepulcre et al., 2010) has been suggested to enhance network information capacity and discrimination accuracy of the global network (Deco et al., 2014). In this regard, the local-to-remote FC has been suggested to provide a comprehensive understanding of AD (Liu et al., 2014; Li et al., 2021). Therefore, the present study assessed the effect of the *APOE* ε4 carrier status by measuring the local and remote FC in an integrative manner. Furthermore, we included information on both global and regional Aβ accumulation and evaluated both memory performance and executive functions being affected at an initial stage for detecting subtle differences in Aβ burden and cognitive function in the earliest course of AD (Buckner, 2004; Farrell et al., 2018). In addition, we hypothesized that the *APOE* ε4 carrier would show a distinct pathway from Aβ deposition to cognitive function *via* differences in the local and remote FC in each CN sub-Aβ and Aβ+ group.

## Materials and Methods

### Participants

A total of 182 CN, consisting of 110 with subthreshold Aβ deposition [CN sub-Aβ group (age range: 55–80 years), 30 subjects in the *APOE* ε4 carrier, 80 subjects in the *APOE* ε*4* non-carrier], 72 with positive Aβ deposition [CN Aβ+ group (age range: 57–84 years), 34 subjects in the *APOE* ε4 carrier, and 38 subjects in the *APOE* ε4 non-carrier], were included in the study. Subjects were recruited from volunteers of the Catholic Aging Brain Imaging database, which contains brain scans of patients who visited the outpatient clinic at the Catholic Brain Health Center, Yeouido St. Mary's Hospital, the Catholic University of Korea, from 2017 to 2021.

### Neuropsychological Assessment

The cognitive function of all subjects was assessed using the Korean version of the Consortium to Establish a Registry for AD (CERAD-K) (Lee et al., 2002). Measurements included assessment in the Korean version of the verbal fluency (VF) test, the 15-item Boston naming test, mini-mental state examination (MMSE-K) (Park, 1989), word list memory (WLM), word list recall (WLR), word list recognition (WLRc), constructional praxis, and constructional recall. In addition, total memory (TM) domain scores were obtained by summing up the scores from the WLM, WLR, and WLRc. The total CERAD-K scores were calculated by summing up all subcategory scores, excluding the MMSE-K score. Additionally, the Stroop Word-Color Interference Test and the Trail Making Test B were used to assess executive functioning (Stroop, 1935; Tombaugh, 2004), along with the VF. Higher Trail Making Test B scores mean lower executive function. Details regarding the usage of specific tests and the reviewing process are described in the Supplementary Material. The inclusion criteria were as follows: (1) unimpaired memory function, quantified by scoring above age-, sex-, and education-adjusted cut-offs on the WLM, WLR, and WLRc domains, (2) MMSE-K score between 24 and 30, (3) Clinical Dementia Rating score of 0, (4) Memory Box score of 0, (5) normal cognitive function based on the absence of significant impairment in cognitive functions or activities of daily living, and (6) participants without a family history of AD. We excluded participants with a history of alcoholism, drug abuse, head trauma, or psychiatric disorders, those taking any psychotropic medications (e.g., cholinesterase inhibitors, antidepressants, benzodiazepines, and antipsychotics), those with multiple vascular risk factors, and those with extensive cerebrovascular disease. T2-weighted fluid-attenuated inversion recovery data were acquired to objectively exclude vascular lesions or other diseases. Participants underwent [^18^F] flutemetamol (FMM) PET-CT within 3 months before or after the magnetic resonance imaging (MRI) scan. The procedures for *APOE* genotyping are described in the Supplementary Material. We excluded participants with the *APOE* ε2 allele. If the participants had at least one *APOE* ε4 allele, they were categorized as *APOE* ε4 carriers; if they had no *APOE* ε4 allele, they were categorized as *APOE* ε4 non-carriers. The study was conducted under the ethical and safety guidelines set forth by the Institutional Review Board of the Catholic University of Korea, which approved all research activities. Informed written consent was obtained from all the participants.

### Functional MRI Data Processing

Detailed procedures for structural and functional MRI (fMRI) data acquisition are described in the Supplementary Material. We used the Data Processing Assistant for resting-state fMRI (rfMRI) (DPARSF, GNU General Public License, Beijing, China) (Yan and Zang, 2010), which is based on the Statistical Parametric Mapping (SPM 12, http://www.fil.ion.ucl.ac.uk/spm, Wellcome Centre for Human Neuroimaging, London, England), to preprocess the fMRI images. The preprocessing included slice timing, realignment for motion corrections, spatial registration, normalization, and smoothing. This procedure is demonstrated in detail in our previous study (Kang et al., 2021) and in the Supplementary Material.

### Local Functional Connectivity Analysis: Regional Homogeneity

Local FC is defined by the temporal coherence or synchronization of the BOLD time series within a set of nearest neighbors of a given voxel. Regional homogeneity (ReHo) is the most representative and reliable index of the local FC (Jiang and Zuo, 2016). The ReHo maps of all participants were made using a general routine using the DPARSF. Briefly, we set the basic cube to calculate Kendall's coefficient of concordance (KCC) by 3 mm × 3 mm × 3 mm voxels. The KCC is obtained from Kendall's rank correlation (Kendall and Gibbons, 1990) and it measures the similarity of the time series of a given voxel to those of its nearest neighbors in a voxel-wise way (Zang et al., 2004). Therefore, the KCC value of the central voxel in the cube was calculated by referring to the temporal sequences of the neighboring 26 voxels. The calculated value was assigned as the ReHo value of the central voxel. To improve the comparability between subjects, standard normal z-transformation was applied to all ReHo maps (zReHo maps). Finally, these “zReHo maps” were spatially smoothed using a 6 mm full width at half maximum Gaussian kernel for the following statistical analysis.

### Remote Functional Connectivity Analysis: Intra- and Inter-Network Connectivity

Regarding the remote FC, we used the default-mode network (DMN) (Sorg et al., 2007; Zhu et al., 2013), central-executive network (CEN) (Weiler et al., 2014), and salience network (SN) (He et al., 2014) that have been demonstrated to be selectively disrupted in the trajectory of AD. In addition, intra- and inter-networks FC of these resting-state networks have been reported to be affected by AD progression (Brier et al., 2012; Wang et al., 2015). Twenty-one spherical (6 mm radius) regions of interest (ROIs) that represented the DMN, CEN, and SN have been described in a previous study (Brier et al., 2012); the Montreal Neurological Institute coordinates of these 21 ROIs are presented in Supplementary Table S1. The representative mean time series were estimated by averaging the time series of all voxels in the ROI. Pearson's correlation coefficients were computed between each pair of ROIs for each subject. Fisher's r-to-z transformation was applied to obtain Z-scores and to improve the normality of the correlation coefficients. For each of the three resting-state networks, the intra-network strength was defined as the mean connection strength of the ROIs in the same network (Supplementary Material). In each pair of networks, the strength of the inter-network connectivity was defined as the mean strength of all possible connections (Supplementary Material).

### [^18^F]-Flutemetamol PET Image Acquisition and Processing

[^18^F] flutemetamol was manufactured, and FMM-PET data were collected and analyzed as described previously (Thurfjell et al., 2014). The MRI of each participant was used to co-register and define the ROIs and correct partial volume effects arising from the expansion of cerebrospinal spaces accompanying the cerebral atrophy. Static PET scans were acquired from 90 to 110 min after 185 MBq of FMM injection. The semi-quantification of FMM uptake on PET/CT scan was performed by obtaining the standardized uptake value ratios (SUVRs). The volumes of interest (VOIs) were restricted to gray matter, covering the frontal, superior parietal, lateral temporal, anterior, and posterior cingulate cortex/precuneus regions. These VOIs were also considered in a previous study (Thurfjell et al., 2014). The reference region for SUVR calculations was pons. The mean uptake counts of each VOIs and reference region were measured on the preprocessed image. A regional SUVR was calculated as the ratio of each cortical regional mean count to the pons mean count (SUVR_PONS_). The global cortical average (composite SUVR) was calculated by averaging the regional cortical SUVRs weighted for size. We used a cut-off for “positive” or “subthreshold” neocortical SUVR of 0.62, consistent with the cut-off values used in a previous FMM PET study (Thurfjell et al., 2014). PET scans classified as subthreshold Aβ accumulation also exhibited normal visual reading. Detailed information on the PET scan and SUVR calculation are provided in the Supplementary Material.

### Statistical Analysis

Statistical analyses were performed using R software (version 2.15.3), jamovi (version 1.6.23) (https://www.jamovi.org), and SPM 12. Assumptions of normality were tested for continuous variables using the Kolmogorov–Smirnov test in R software; all data demonstrated a normal distribution. The two-sample *t*-test and chi-squared (χ^2^) tests were used to probe for differences in demographic variables, clinical data, regional and global Aβ deposition, and cognitive function between *APOE* ε4 carriers and non-carriers in each CN sub-Aβ and Aβ+ group, respectively. All statistical analyses used a two-tailed *P-*value < 0.05 to define statistical significance.

A multiple regression analysis was applied to investigate the effects of *APOE* ε4 carrier status-by-Aβ accumulation interactions on the neuropsychological test scores in each CN sub-Aβ and Aβ+ group. CERAD-K subdomain, TM, and total CERAD-K scores were the dependent variables, while *APOE* ε4 carrier status, regional, and global FMM SUVR_PONS_ were the independent variables. Age, sex, and years of education were included as covariates. We applied a threshold of α = 0.05 to consider significant regression weights, and we additionally accounted for multiple testing using the Bonferroni correction for each hypothesis (multiplying the *P*-value by a factor of 12 subdomains of the CERAD-K battery). In addition, each variable was z-transformed using the mean and standard deviation for further analysis.

To compare the difference in the local FC depending on the *APOE* ε4 carrier status, ANCOVA on a voxel-by-voxel basis was carried out between the *APOE* ε4 carriers and non-carriers on the individual z maps of ReHo in each CN sub-Aβ and Aβ+ group. Age, sex, and years of education were included as covariates in the statistical tests. We designed an ANCOVA based on SPM 12. All statistical maps were corrected for multiple comparisons using Gaussian random field (GRF) correction combining the voxel *P*-value < 0.001 and cluster level <0.05 in DPABI_V5.1_201201 (http://rfmri.org/dpabi, GNU GENERAL PUBLIC LICENSE, Beijing, China) (Bansal and Peterson, 2018). This cluster-wise method based on the random field theory is recommended for multiple comparisons of ReHo (Jiang and Zuo, 2016). In addition, zReHo values from brain regions with significant group differences were used for further ROI analysis.

We performed multiple regression analysis to evaluate the impact of the ReHo-by-*APOE* ε4 carrier status interaction on neuropsychological test scores. CERAD-K subdomain, TM, and total CERAD-K scores were the dependent variables, while *APOE* ε4 carrier status and zReHo values from the brain regions with significant group differences were the independent variables. Age, sex, and years of education were included as covariates. A Bonferroni correction was performed for the 12 subdomains of the CERAD-K battery (multiplying the *P*-value by a factor of 12). A significance threshold of 0.05 was used.

Furthermore, a general linear model (GLM) based on whole-brain analysis was performed on the individual z maps of ReHo to evaluate the impact of Aβ deposits-by-*APOE* ε4 carrier status interaction on the local FC in each CN sub-Aβ and Aβ+ group. The *APOE* ε4 carrier status, regional, and global FMM SUVR_PONS_ were the independent variables. We controlled the effects of age, sex, and years of education using GLM analysis implemented in SPM 12. The threshold was set at *P* < 0.05 [false discovery rate (FDR)] to control multiple comparisons (Genovese et al., 2002).

Additionally, we applied a multiple regression analysis to examine the impact of *APOE* ε4 carrier status-by-local FC interactions on the remote FC in each CN sub-Aβ and Aβ+ group. The dependent variables were mean z-transformed correlation values within and between the DMN, CEN, and SN, while independent variables were *APOE* ε4 carrier status and zReHo values from the brain regions with significant differences between *APOE* ε4 carriers and non-carriers. Age, sex, and years of education were included as covariates.

Finally, we performed a multiple regression analysis to evaluate the effect of *APOE* ε4 carrier status-by-remote FC interactions on the cognitive function in each CN sub-Aβ and Aβ+ group. The CERAD-K subdomain, TM, and total CERAD-K scores were the dependent variables, while *APOE* genotype and mean z-transformed correlation values within and between the DMN, CEN, and SN were the independent variables. Age, sex, and years of education were included as covariates. We applied a threshold of α = 0.05, and 0.005 to consider significant regression weights. A Bonferroni correction was additionally performed for multiple comparisons (multiplying the *P*-value by a factor of six intra- and inter-network FC). Statistical significance was set at a Bonferroni-corrected *P* < 0.05.

Furthermore, to exclude the effect of cortical atrophy on local and remote FC, we conducted a whole-brain voxel-wise analysis of between-group differences (*APOE* ε4 carrier vs. non-carrier) in gray matter volume with a GLM using SPM12, controlling for age, sex, years of education, and intracranial volume in each CN sub-Aβ and Aβ+ group (Supplementary Material).

## Results

### Baseline Demographic and Clinical Data

Tables 1A,B show the baseline demographic data for the CN sub-Aβ and Aβ+ groups. There were no significant differences in age, sex, and the number of years of education between *APOE* ε4 carriers and non-carriers in either group. Regarding the global and regional FMM SUVR_PONS_, no significant differences were found between *APOE* ε4 carriers and non-carriers in each CN sub-Aβ and Aβ+ group. In neuropsychological test scores, *APOE* ε4 non-carriers showed significantly higher scores in the CERAD-K BNT subdomains than the *APOE* ε4 carriers in the CN sub-Aβ group (*P*-value < 0.001). However, the remaining domains, TM, and total scores showed no significant difference between *APOE* ε4 carriers and non-carriers in both CN sub-Aβ and Aβ+ groups.

**Table 1:** Demographic and clinical characteristics of study participants.

**(A) Cognitively normal older adults with subthreshold Aβ** **deposition**.			
*APOE* ε4 carrier status	**Non-carrier**	**Carrier**	***P*** **-value**
	**(*****n*** **=** **80)**	**(*****n*** **=** **30)**	
Age	67.0 ± 6.1	67.3 ± 7.7	0.850
Gender		
Male	23 (28.8%)	10 (33.3%)	
Female	57 (71.2%)	20 (66.7%)	
Education years	12.8 ± 3.6	12.7 ± 4.1	0.951
Global SUVR_PONS_	0.56 ± 0.03	0.55 ± 0.04	0.204
**Regional SUVR**_**PONS**_			
ACC	0.57 ± 0.04	0.58 ± 0.05	0.323
FL	0.44 ± 0.04	0.43 ± 0.04	0.230
PL	0.37 ± 0.04	0.38 ± 0.06	0.563
PCC/precuneus	0.49 ± 0.04	0.48 ± 0.04	0.385
TL	0.51 ± 0.03	0.51 ± 0.04	0.768
**CERAD-K**			
VF	17.0 ± 4.2	15.5 ± 3.1	0.070
BNT	13.2 ± 1.4	12.2 ± 1.7	0.001
MMSE	28.3 ± 1.4	28.2 ± 1.3	0.989
WLM	20.2 ± 3.2	20.3 ± 3.0	0.911
CP	10.8 ± 0.6	10.8 ± 0.8	0.976
WLR	7.0 ± 1.5	6.9 ± 1.3	0.750
WLRc	9.5 ± 0.7	9.5 ± 0.8	0.651
CR	8.4 ± 2.6	7.3 ± 2.8	0.055
TM	36.8 ± 4.7	36.7 ± 4.2	0.922
Total	86.2 ± 9.9	82.3 ± 9.1	0.063
TMT B	118.2 ± 61.6	139.8 ± 68.6	0.115
Stroop word-color	41.7 ± 10.8	39.8 ± 8.5	0.373
**(B) Cognitively normal older adults with positive Aβ** **deposition**			
*APOE* ε4 carrier status	**Non-carrier**	**Carrier**	***P*** **-value**
	**(*****n*** **=** **38)**	**(*****n*** **=** **34)**	
Age	74.5 ± 6.6	72.5 ± 7.8	0.249
Gender			1.000
Male	11 (28.9%)	10 (29.4%)	
Female	27 (71.1%)	24 (70.6%)	
Education years	10.8 ± 4.6	11.2 ± 5.1	0.700
Global SUVR_PONS_	0.75 ± 0.09	0.72 ± 0.07	0.114
**Regional SUVR**_**PONS**_			
ACC	0.74 ± 0.09	0.72 ± 0.09	0.319
FL	0.67 ± 0.11	0.64 ± 0.10	0.276
PL	0.58 ± 0.11	0.53 ± 0.08	0.078
PCC/precuneus	0.77 ± 0.14	0.72 ± 0.12	0.088
TL	0.69 ± 0.10	0.65 ± 0.09	0.082
**CERAD-K**			
VF	14.0 ± 4.1	15.1 ± 4.2	0.267
BNT	11.9 ± 2.2	12.1 ± 2.0	0.616
MMSE	26.7 ± 2.8	27.3 ± 2.0	0.357
WLM	17.3 ± 3.7	17.5 ± 3.8	0.802
CP	10.3 ± 1.2	10.4 ± 1.1	0.631
WLR	5.5 ± 1.7	5.5 ± 2.0	0.871
WLRc	9.0 ± 0.9	9.2 ± 0.9	0.329
CR	5.7 ± 3.0	6.6 ± 2.5	0.165
TM	31.8 ± 5.6	32.3 ± 5.9	0.707
Total	73.5 ± 13.2	76.6 ± 12.0	0.306
TMT B	196.6 ± 87.3	177.5 ± 80.4	0.338
Stroop word-color	31.5 ± 8.0	34.6 ± 13.3	0.249

*Data are presented as the mean ± standard deviation unless indicated otherwise. SUVR_PONS_, standardized uptake value ratios of [^18^F] flutemetamol (FMM), using the pons as a reference region; ACC, anterior cingulate cortex; FL, frontal lobes; PL, parietal lobes; PCC/precuneus, Posterior cingulate cortex and precuneus; TL, lateral temporal lobes; CERAD-K, Korean version of the Consortium to Establish a Registry for Alzheimer's Disease; VF, verbal fluency; BNT, Boston Naming Test; MMSE, Korean version of the Mini Mental Status Examination; WLM, word list memory; CP, constructional praxis; WLR, word list recall; WLRc, word list recognition; CR, constructional recall; TM, total scores of memory domains, including CERAD-K WLM, WLR, WLRc; Total, total scores of CERAD-K VF, BNT, WLM, CP, WLR, WLRc, and CR domains; TMT B, trail making test B*.

### Association Between the Quantitative Value of Aβ Accumulation and Neuropsychological Test Scores According to *APOE* ε4 Carrier Status

**Figure 1:**
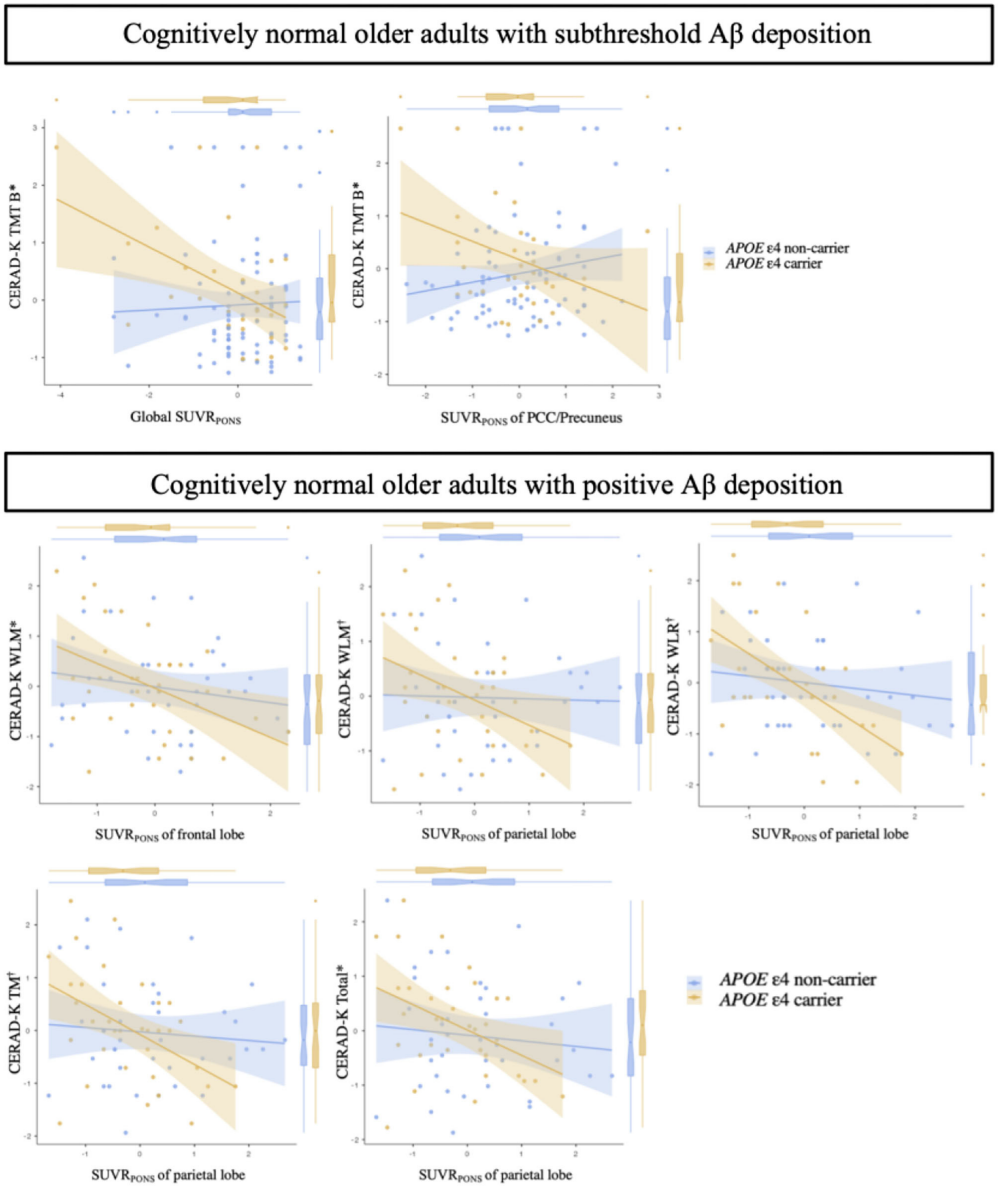
Association between the quantitative value of Aβ accumulation and neuropsychological test scores in cognitively normal older adults with subthreshold and positive Aβ deposition according to the *APOE* ε4 carrier status. *, uncorrected *P* < 0.05;, Bonferroni corrected *P* < 0.05; Aβ deposition-by-*APOE* ε4 carrier status interaction for neuropsychological test scores by multiple regression analysis adjusting for age, sex, and years of education. Cognitively normal older adults with subthreshold Aβ deposition: Global SUVR_PONS_, adjusted R^2^ = 0.552; PCC/precuneus SUVR_PONS_, adjusted R^2^ = 0.533. Cognitively normal older adults with positive Aβ deposition: WLM-frontal lobe SUVR_PONS_, adjusted R^2^ = 0.531; WLM-parietal lobe SUVR_PONS_, adjusted R^2^ = 0.549; WLR-parietal lobe SUVR_PONS_, adjusted R^2^ = 0.358; TM-parietal lobe SUVR_PONS_, adjusted R^2^ = 0.518; Total-parietal lobe SUVR_PONS_, adjusted R^2^ = 0.503. Each variable was z-transformed using the mean and standard deviation. CERAD-K, Korean version of the Consortium to Establish a Registry for Alzheimer's Disease; SUVR_PONS_, standardized uptake value ratios of [^18^F] flutemetamol (FMM), using the pons as a reference region; PCC/precuneus, posterior cingulate cortex and precuneus; TM, total scores of memory domains; TMT B, trail making test B; WLM, word list memory; WLR, word list recall.

Figure 1 displays the differential associations between the regional and global Aβ accumulation and cognitive performance scores depending on the *APOE* ε4 carrier status in each CN sub-Aβ and Aβ+ group. In the CN sub-Aβ group, there was a significant interaction between *APOE* ε4 carrier status and Aβ deposits such that the executive function was higher in the *APOE* ε4 carrier that showed higher global and regional FMM SUVR_PONS_ in the PCC/precuneus (uncorrected *P* < 0.05), yielding a large effect size. In the CN Aβ+ group, there was an interaction between the *APOE* ε4 carrier status and the regional FMM SUVR_PONS_ in the frontal and parietal lobes for WLM, WLR, TM, and CERAD-K total scores (uncorrected *P* < 0.05, WLM-frontal lobe SUVR_PONS_, and CERAD-K total-parietal lobe SUVR_PONS_; Bonferroni corrected *P* < 0.05, WLM-parietal lobe SUVR_PONS_, WLR-parietal lobe SUVR_PONS_, and TM-parietal lobe SUVR_PON_). Lower memory and global performances were displayed in the *APOE* ε4 carriers with higher regional Aβ accumulation.

### Brain Regions Showing Differences in Local Connectivity Between *APOE* ε4 Carriers and Non-carriers

**Figure 2:**
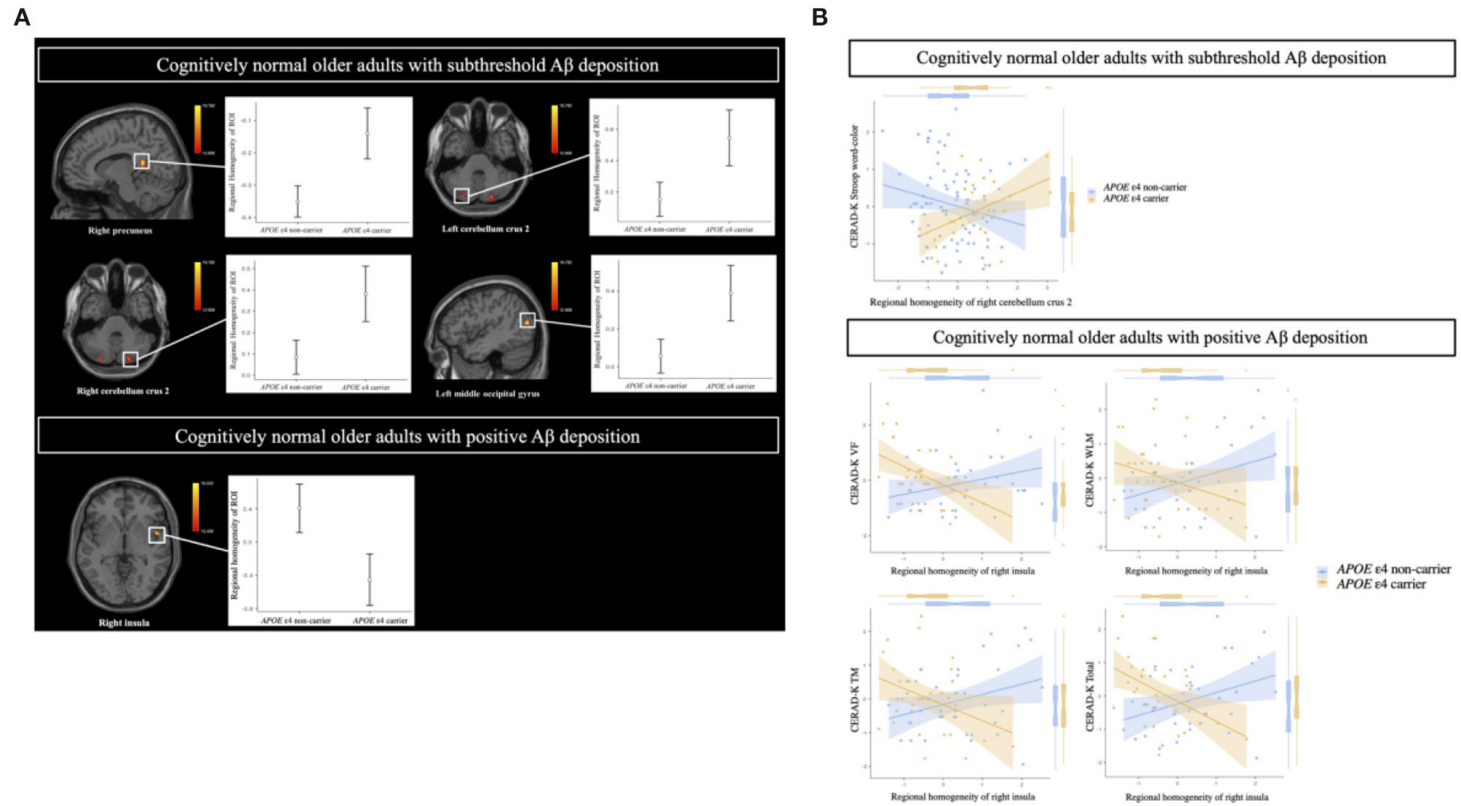
**(A)** Brain regions showing a significant difference in the regional homogeneity in cognitively normal older adults with subthreshold and positive Aβ deposition between *APOE* ε4 carriers and non-carriers. **(B)** Relationships of cognitive function to regional homogeneity in regions of interest (ROIs) according to *APOE* ε4 carrier status in cognitively normal older adults with subthreshold and positive Aβ deposition. **(A)** General linear model (GLM) analysis adjusted for age, sex, and years of education. Thresholds are set using GRF correction at a *P*-value of < 0.05, voxel *P* < 0.001. Cognitively normal older adults with subthreshold Aβ deposition: The statistical threshold of the cluster size > 35; cognitively normal older adults with positive Aβ deposition: The statistical threshold of the cluster size > 29. **(B)** Multiple regression analysis adjusted for age, sex, and education years (Bonferroni corrected *P* < 0.05). Each variable was z-transformed using the mean and standard deviation. CERAD-K, Korean version of the Consortium to Establish a Registry for Alzheimer's Disease; VF, verbal fluency; WLM, word list memory; TM, total scores of memory domains, including CERAD-K WLM, WLR, and WLRc.

In the CN sub-Aβ group, *APOE* ε4 carriers exhibited significantly higher ReHo than *APOE* ε4 non-carriers did in the right precuneus, the left middle occipital gyrus, and the bilateral cerebellum crus 2 (GRF correction at a *P*-value of < 0.05, voxel *P* < 0.001). Table 2A and Figure 2A provide an overview of the differences in the local connectivity. However, as can be seen from Table 2B and Figure 2A, the CN Aβ+ group with the *APOE* ε4 carrier status showed significantly lower ReHo in the right insula than did the *APOE* ε4 non-carriers. Additionally, there was a significant interaction between the *APOE* ε4 carrier status and ReHo in the ROIs for the neuropsychological test scores in both the CN sub-Aβ and Aβ+ groups (Bonferroni corrected *P* < 0.05). As expected from the visual inspection of the results shown in Figure 2B, higher ReHo in the right cerebellum crus 2 was associated with higher executive function in the *APOE* ε4 carrier of the CN sub-Aβ group, but lower ReHo in the right insula was correlated with higher memory and executive function in the *APOE* ε4 carrier of the CN Aβ+ group.

**Table 2:** Anatomical locations of regions showing a significant difference in the regional homogeneity for cognitively normal older adults with (A) subthreshold and (B) positive Aβ deposition between *APOE* ε4 carriers and non-carriers.

**(A)**						
**Region**	**L/R**	**Cluster** **(Voxel count)**	**Peak F value**	**Peak MNI coordinates** **(x, y, z)**		
**Significant difference in ReHo between** ***APOE*** **ε4 carriers and non-carriers**						
Precuneus	R	66	19.1519	−10	−46	12
Cerebellum Crus 2	L	47	14.6207	−26	−82	−34
Cerebellum Crus 2	R	55	16.5878	22	−84	−36
Middle occipital gyrus	L	45	19.7821	−44	−76	14
**(B)**						
**Region**	**L/R**	**Cluster** **(Voxel count)**	**Peak F value**	**Peak MNI coordinates** **(x, y, z)**		
**Significant difference in ReHo between** ***APOE*** **ε4 carriers and non-carriers**						
Insula	R	33	19.3333	48	2	−4

*General linear model (GLM) analysis adjusted for age, sex, and years of education Thresholds were set using GRF correction at a p-value of < 0.05, voxel P < 0.001. The statistical threshold of (A) cluster size > 35, (B) cluster size > 29. L, left; R, right; ReHo, regional homogeneity*.

### *APOE* ε4 Carrier Status-by-Aβ Deposition Interaction for Local Connectivity

**Figure 3:**
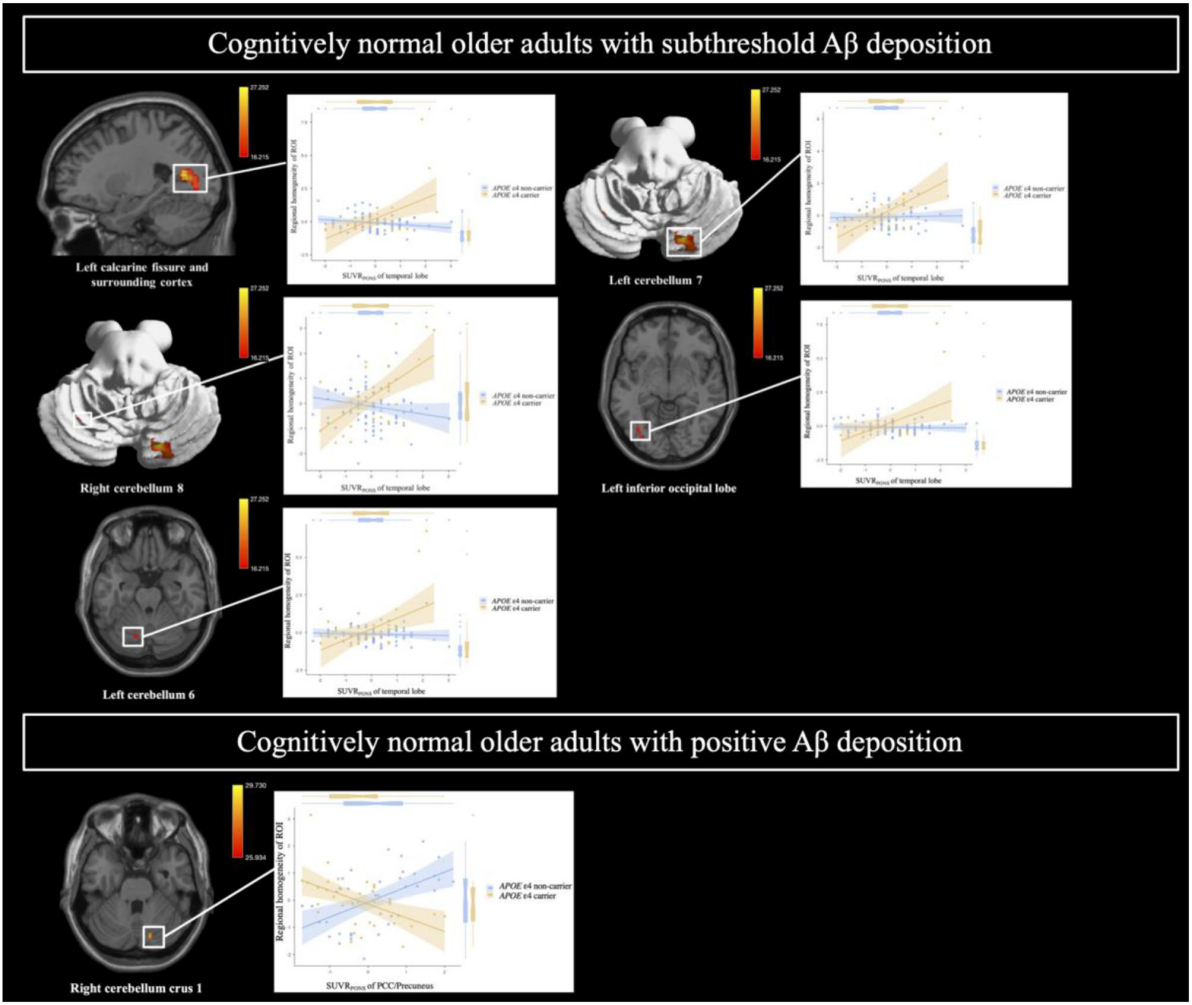
Brain regions showing a significant interaction between Aβ deposition and *APOE* ε4 carrier status for regional homogeneity in cognitively normal older adults with subthreshold and positive Aβ deposition. General linear model (GLM) analysis adjusted for age, sex, and years of education (FDR-adjusted *P* < 0.05). Each variable was z-transformed using the mean and standard deviation. SUVR_PONS_, standardized uptake value ratios of [^18^F] flutemetamol (FMM).

After adjusting for age, sex, and years of education, the Aβ deposition-by-*APOE* ε4 carrier status interaction demonstrated a significant effect on ReHo in both CN sub-Aβ and Aβ+ groups (FDR-adjusted *P* < 0.05). In the CN sub-Aβ group, correlation slopes between the temporal lobe Aβ accumulation and ReHo of the ROIs were more positive for *APOE* ε4 carriers than for non-carriers (Figure 3). However, in the CN Aβ+ group, lower ReHo of the right cerebellum crus 1 was shown in the *APOE* ε4 carrier with higher regional Aβ deposition in the PCC/precuneus (Figure 3). These anatomical locations, their corresponding MNI coordinates, and the intensities of the peak points in each cluster are shown in Table 3.

**Table 3:** Anatomical locations of regions showing a significant interaction between Aβ deposition and *APOE* ε4 carrier status for regional homogeneity in cognitively normal older adults with (A) subthreshold and (B) positive Aβ deposition.

**(A)**						
**Region**	**L/R**	**Cluster** **(Voxel count)**	**Peak** ***F*****-value**	**Peak MNI coordinates** **(x, y, z)**		
**Temporal lobe SUVR**_**PONS**_**-by-*****APOE*** **ε4 carrier status interaction for regional homogeneity**						
Calcarine fissure and surrounding cortex	L	323	27.2528	−26	−64	12
Cerebellum 7	L	148	26.3141	−14	−76	−46
Cerebellum 8	R	50	23.5239	24	−64	−54
Inferior occipital lobe	L	49	19.5212	−36	−70	−4
Cerebellum 6	L	34	17.874	−18	−66	−24
**(B)**						
**Region**	**L/R**	**Cluster** **(Voxel count)**	**Peak F value**	**Peak MNI coordinates** **(x, y, z)**		
**PCC/precuneus SUVR**_**PONS**_**-by-*****APOE*** **ε4 carrier status interaction for regional homogeneity**						
Cerebellum Crus 1	R	23	29.7307	16	−78	−24

*General linear model (GLM) analysis adjusted for age, sex, years of education, FDR-adjusted P < 0.05, SUVR_PONS_, standardized uptake value ratios of [^18^F] flutemetamol (FMM); L, left; R, right; PCC/precuneus, posterior cingulate cortex and precuneus; SUVR_PONS_, standardized uptake value ratios of [^18^F] FMM, using the pons as a reference region*.

### Impact of Local Connectivity on Remote Connectivity Depending on *APOE* ε4 Carrier Status

**Figure 4:**
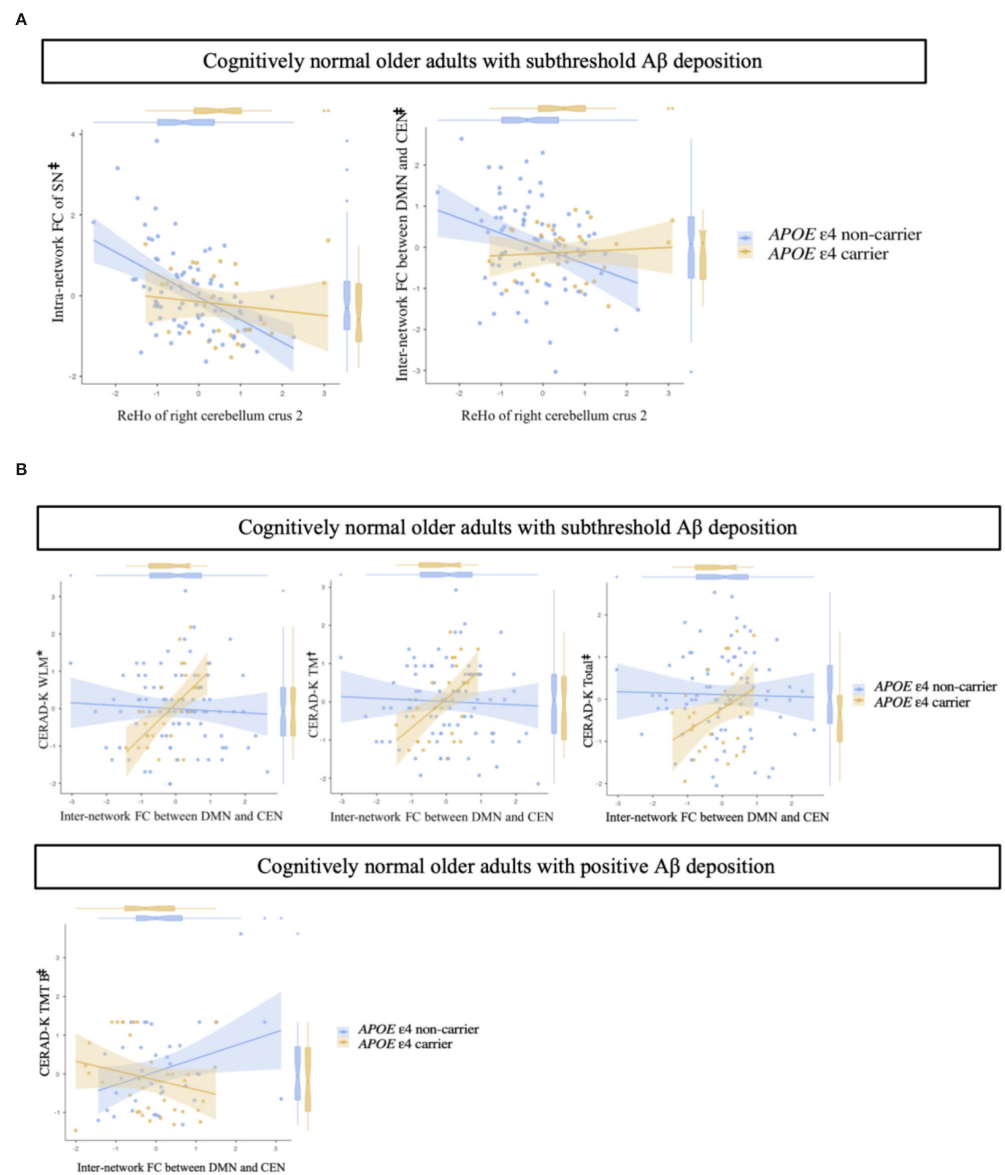
**(A)** Impact of regional homogeneity on intra- and inter-network functional connectivity in cognitively normal older adults with subthreshold Aβ deposition according to *APOE* ε4 carrier status. **(B)** Interaction of inter-network functional connectivity with *APOE* ε4 carrier status for memory performances and executive function in cognitively normal older adults with subthreshold and positive Aβ deposition. Multiple linear regression analysis adjusted for age, sex, and years of education. Each variable was z-transformed using the mean and standard deviation. **(A)** Intra-network FC of the SN, adjusted R^2^ = 0.191; inter-network FC between DMN and CEN, adjusted R^2^ = 0.110. **(B)** Cognitively normal older adults with subthreshold Aβ deposition: WLM, adjusted R^2^ = 0.376; TM, adjusted R^2^ = 0.393; Total, adjusted R^2^ = 0.521. Cognitively normal older adults with positive Aβ deposition: TMT B, adjusted R^2^ = 0.365. *, Bonferroni corrected *P* < 0.05;, uncorrected *P* < 0.005; ^‡^, uncorrected *P* < 0.05. FC, functional connectivity; SN, salience network; DMN, default-mode network; CEN, central-executive network; CERAD-K, the Korean version of the Consortium to Establish a Registry for Alzheimer's Disease; WLM, word list memory; TM, total scores of memory domains; total, total scores summing up all subcategory scores, excluding the MMSE score; TMT B, trail making test B.

In terms of intra- and inter-network remote FC between the DMN, CEN, and SN, there were no significant differences between *APOE* ε4 carriers and non-carriers in the CN sub-Aβ and Aβ+ groups (Bonferroni corrected *P* > 0.05, Supplementary Figures 1, 2). In addition, for the intra-network FC of the SN and the inter-network FC between the DMN and CEN, only the CN sub-Aβ group exhibited a significant interaction between the *APOE* ε4 carrier status and the local connectivity in the right cerebellum crus 2, in which higher local connectivity was found in the *APOE* ε4 carrier than in the non-carrier group (uncorrected *P* < 0.05, Figure 4A). Although these interactions showed a large effect size, none of the interactions survived Bonferroni correction for multiple comparisons. In addition, we found a significant interaction between the *APOE* ε4 carrier status and the inter-network FC between the DMN and CEN for neuropsychological performance in each CN sub-Aβ and Aβ+ group (Bonferroni corrected *P* < 0.05, WLM; uncorrected *P* < 0.005, TM; uncorrected *P* < 0.05, CERAD-K total and TMT B). In the CN sub-Aβ group, higher memory and global performance were exhibited in the *APOE* ε4 carrier with a stronger inter-network FC between the DMN and CEN. In the CN Aβ+ group, a higher executive function was found in the *APOE* ε4 carrier with a stronger inter-network FC between the DMN and CEN. Figure 4B provides an overview of these interactions for neuropsychological performance.

## Discussion

In the current study, we found a distinctive association between the regional, global Aβ accumulation, and cognitive performance scores depending on the *APOE* ε4 carrier status in each CN sub-Aβ and Aβ+ group. In the CN sub-Aβ group, the executive function was higher in the *APOE* ε4 carrier that showed higher global and regional Aβ accumulation. However, lower memory and global performances were displayed in the *APOE* ε4 carriers with higher regional Aβ deposition. Taken together, these results show that the area of cognitive function and the slope of plots between the Aβ pathology and cognitive function differed according to the degree of Aβ deposits in the *APOE* ε*4* carrier in the preclinical phase. This result may also reflect the compensatory reaction in the executive function against the subthreshold Aβ accumulation in the CN. Additionally, we found a significant relationship between cognitive function and Aβ deposits in the posterior cortical regions. This finding broadly supports the work of a previous study demonstrating a more accurate prediction of prospective cognitive decline by Aβ accumulation in the posterior brain areas than by global deposits (Farrell et al., 2018). However, this previous study could not confirm the significant impact of the *APOE* ε4 allele on the results. In another study, a change in the subthreshold Aβ deposition was associated with memory decline but not executive function (Landau et al., 2018). In this prior study, the average age of the participants was older than that in the current study, and the presence of the *APOE* ε4 allele was not adjusted for the analysis. Moreover, the proportion of *APOE* ε4 carriers was only 15%. Therefore, such differences may have caused the discrepancy between the studies. Finally, the current study evaluated the association between Aβ accumulation and subtle cognitive decline within the normal range, which might have contributed to the relative lack of statistical robustness for the interaction.

Regarding the local FC, the *APOE* ε4 carriers exhibited significantly higher ReHo, mainly in the brain region of the DMN in the CN sub-Aβ group. However, there is little published research on the impact of the *APOE* ε4 allele on local FC in the preclinical phase of AD. Given the positive relationship between ReHo and regional glucose metabolism (Nugent et al., 2015), *APOE* ε4 carriers display increased glucose metabolism in the medial, frontal, and anterior temporal regions of the CN (Yi et al., 2014). However, this previous study did not evaluate Aβ deposition, and it assessed the impact of the *APOE* ε4 allele by including this high-risk genotype as the covariate in the analysis with small sample size. Another study with subjects complaining of subjective cognitive decline (SCD) showed a significant difference in the ReHo according to the Aβ status. However, this previous study did not explore the interaction with the *APOE* ε4 allele; it included subjects who were older than those in the present study, and also performed the analysis in the restricted sample size (Li et al., 2021). These previous results, therefore, need to be interpreted with caution regarding possible bias.

Among the ROIs showing a significantly higher ReHo in the *APOE* ε4 carrier of the CN sub-Aβ group, the precuneus is the known hub region of the DMN, and the ReHo in this region reflects the progression of AD (He et al., 2007; Kang et al., 2017). Although prior research has documented the negative relationship between Aβ deposition and ReHo of the precuneus in the CN with a positive Aβ PET scan, another previous study has demonstrated that SCD subjects with Aβ deposits show a higher ReHo in the right precuneus than those without Aβ accumulation, suggesting the compensatory role of the increased local FC (Li et al., 2021). However, given the Aβ-dependent neuronal hyperactivation (Zott et al., 2019), we could not rule out the possibility that early Aβ deposition induced a higher ReHo in the AD vulnerable brain region.

However, despite the importance of the *APOE* ε4 allele, there remains a paucity of evidence on the effect of the *APOE* ε4 allele on the local FC change in the earliest phase of AD. Concerning the middle occipital gyrus, which is another ROI in the CN sub-Aβ group, the FC between this ROI and precuneus has been shown to be higher in SCD subjects with Aβ burden than in those without Aβ deposits (Li et al., 2021). Additionally, we found a higher ReHo in several posterior regions of the cerebellum in the *APOE* ε4 carriers in the CN sub-Aβ group. The posterior cerebellum has been reported to be functionally mapped to the DMN brain regions (Buckner et al., 2011; Buckner, 2013) that are prone to Aβ accumulation (Mormino et al., 2011). In this regard, the Aβ-associated neuronal hyperactivation might affect the high activity of the DMN brain regions and functionally related posterior cerebellum (Pasquini et al., 2017). Additionally, neuronal hyperactivity has also been reported to induce further Aβ deposition (Bero et al., 2012). This positive correspondence between Aβ and intrinsic FC has been demonstrated to start in the preclinical stage (Pasquini et al., 2017). Additionally, a previous study has also shown the impact of the *APOE* ε4 allele on neuronal hyperactivity in a mouse model without definite Aβ pathology (Nuriel et al., 2017). Therefore, a synergistic effect of the *APOE* ε4 allele with the Aβ burden could affect the high activity of the ROIs in the current study. However, the posterior cerebellum did not show a significant difference depending on the *APOE* ε4 carrier status in the CN Aβ+ group of the present study. The detrimental effect of Aβ on FC has been reported to appear in brain networks where both the brain activity and Aβ deposits are high (Pasquini et al., 2017). In addition, this deteriorating effect has been reported to initiate already in the preclinical phase and reach a peak in the MCI stage (Pasquini et al., 2017). In this regard, a higher Aβ burden might weaken the brain activity of the ROIs in the CN Aβ+ group. However, a further longitudinal study is required to determine exactly how Aβ influences the brain functional change, conjoining with *APOE* ε4 allele in the preclinical phase.

In the CN Aβ+ group in the present study, the *APOE* ε4 carriers showed significantly lower ReHo in the insula, which is a known hub region of the SN. The insula has been demonstrated to have a modulatory role in the association between the DMN and CEN (Sridharan et al., 2008). Additionally, the SN has been documented to display distinctive FC activation from normal aging to AD progression (He et al., 2014). Contrary to the current findings, the *APOE* ε4 carrier in the preclinical phase has demonstrated increased FC related to the insula, which is attributed to the reduction of the inhibitory control of the DMN (Machulda et al., 2011). However, in the present study, there were no significant differences in the intra-network FC of the DMN and the inter-network FC between the DMN and SN depending on the *APOE* ε4 carrier status in the CN Aβ+ group. Nevertheless, caution should be exercised here because there was a lack of information on Aβ deposition in the above-mentioned previous studies, and the difference in the ReHo has been shown depending on Aβ positivity during the preclinical phase (Kang et al., 2017).

In the present study, the difference in ReHo in the *APOE* ε4 carriers was related to the higher executive and memory function in each CN sub-Aβ and CN Aβ+ group, respectively. Additionally, this finding broadly supports the work of other studies linking the functional activation of the posterior cerebellum with executive function (Baumann et al., 2015; Castellazzi et al., 2018). In this regard, this combination of findings provides some support for the compensatory role of the difference in the ReHo found in the *APOE* ε4 carriers in the preclinical phase. However, these results differ from some published studies reporting a non-significant association between FC and cognitive function in *APOE* ε4 carriers with intact cognition (Chen et al., 2015). These differences can be explained in part by not considering the effect of the Aβ burden in the previous study.

Another interesting finding was the differential association between regional Aβ burden and local connectivity according to the *APOE* ε4 carrier status in each CN sub-Aβ and Aβ+ group. In the *APOE* ε4 carriers in the CN sub-Aβ group, the ReHo was higher in ROI, including the posterior cerebellum, as the Aβ accumulation in the temporal lobe was higher, but in the CN Aβ+ group, the ReHo was lower in the right cerebellum crus 1 as the Aβ burden in the PCC/precuneus was higher. Although the subjects of this study were not in the age group in which Aβ deposition was actively increasing (Rodrigue et al., 2012), the temporal lobe and PCC/precuneus have been reported as early Aβ deposition regions (Sojkova et al., 2011). These regions have been demonstrated to be vulnerable to the *APOE* ε4 allele (Sheline et al., 2010) and to show an overlap between the topological distribution of Aβ deposits and the DMN (Kang et al., 2017). Additionally, hyperactivity in these regions has been assumed to induce further Aβ accumulation, which could cause neuronal dysfunction and reduced FC in the CN (Bero et al., 2011, 2012). In addition, among the ROIs in the current findings, the posterior cerebellum has been demonstrated to be functionally connected to the temporal gyrus and precuneus, which are the vulnerable regions in AD (Guo et al., 2016). Significantly, the cerebellum crus 1 and cerebellum 7 have also been mapped to the DMN brain regions (Buckner et al., 2011), including the association cortex where Aβ accumulation is prone to occur (Mormino et al., 2011). However, few previous studies on the distinctive effect of the *APOE* ε4 allele on the function of the posterior cerebellum have been performed. Therefore, starting from this study, additional reproducible studies are needed to verify the differential effects of the *APOE* ε4 allele. Furthermore, the temporal gyrus has also been known to be correlated with tau pathology (Insel et al., 2020) and a synergistic effect between Aβ and tauopathy for the changes in the FC has been reported (Schultz et al., 2017). Therefore, additional studies evaluating tauopathy are necessary to understand the earliest pathophysiology of AD more accurately.

In the CN sub-Aβ group of the present study, there was a significant interaction between the *APOE* ε4 carrier status and local FC in the right cerebellum crus 2 for the remote inter-network FC between the DMN and CEN, showing robustness to the decline in this inter-network FC in the *APOE* ε4 carriers. This is consistent with previous findings showing a significant association between the posterior cerebellum and resting-state networks, including the DMN and CEN (Habas et al., 2009; Buckner et al., 2011). The inter-network FC between the DMN and CEN has been reported to display a U-shaped progression pattern with increasing age, and to show a declining pattern in the age group of the subjects in the current study (Ng et al., 2016). In this regard, the *APOE* ε4 carrier status could be suggested to have an alleviating role for this declining pattern in the CN before the Aβ positivity. Furthermore, we found that the stronger inter-network FC of DMN-CEN was associated with better cognitive function in the *APOE* ε4 carrier in both CN sub-Aβ and Aβ+ groups. The function of the CEN has been demonstrated to be supported by that of the DMN in maintaining a normal cognition (Turner and Spreng, 2015). In this respect, the present results support previous observational studies that have shown a positive association between the cognitive function and the inter-network FC of DMN-CEN in the cognitively normal *APOE* ε4 carrier of similar age to the present study (Ng et al., 2018).

In the current study, the participants with varying risk for AD have not yet been confirmed to have developed further Aβ accumulation or converted to symptomatic disease. Moreover, considering the results of previous studies showing that Aβ changes rather than that baseline accumulation predicts prospective cognitive change (Farrell et al., 2018); accordingly, it is necessary to conduct a longitudinal study for a clear interpretation of the results of the current study. Given that prior study has demonstrated an interaction between Aβ and tau for changes in the FC of the CN (Hasani et al., 2021), another issue with this study is the lack of information on the tau deposits. Additionally, it has been reported that the FMM has a relatively low sensitivity to diffuse plaques that are usually detected in the earliest phase of Aβ accumulation (Salloway et al., 2017). In this respect, the current findings must be interpreted with caution when comparing them with the results of previous studies using other types of Aβ ligands. In addition, a selection bias is another potential limitation. The *APOE* ε4 carriers whose cognitive function remained intact despite the Aβ burden could have other characteristics, including protective genes (Seto et al., 2021) and higher cognitive reserve (Stern, 2009). The characteristics of these participants might have influenced the non-significant difference in cognitive function according to *APOE* ε4 carrier status. Finally, in the present study, multiple analyzes were performed after dichotomization according to Aβ positivity due to many analysis variables. Additionally, conventional data-fusion methods have difficulty in capturing complex relationships between multiple data, including brain imaging, genetic, demographic, and clinical data (Hu et al., 2019). In this regard, we need to apply deep collaborative learning, which can link the whole data simultaneously to develop a complete understanding of the Aβ pathophysiological cascade during the preclinical phase (Song and Chai, 2018; Hu et al., 2019). This novel method has been demonstrated to improve the generalization and robustness of brain research (Song and Chai, 2018).

## Conclusion

**Figure 5:**
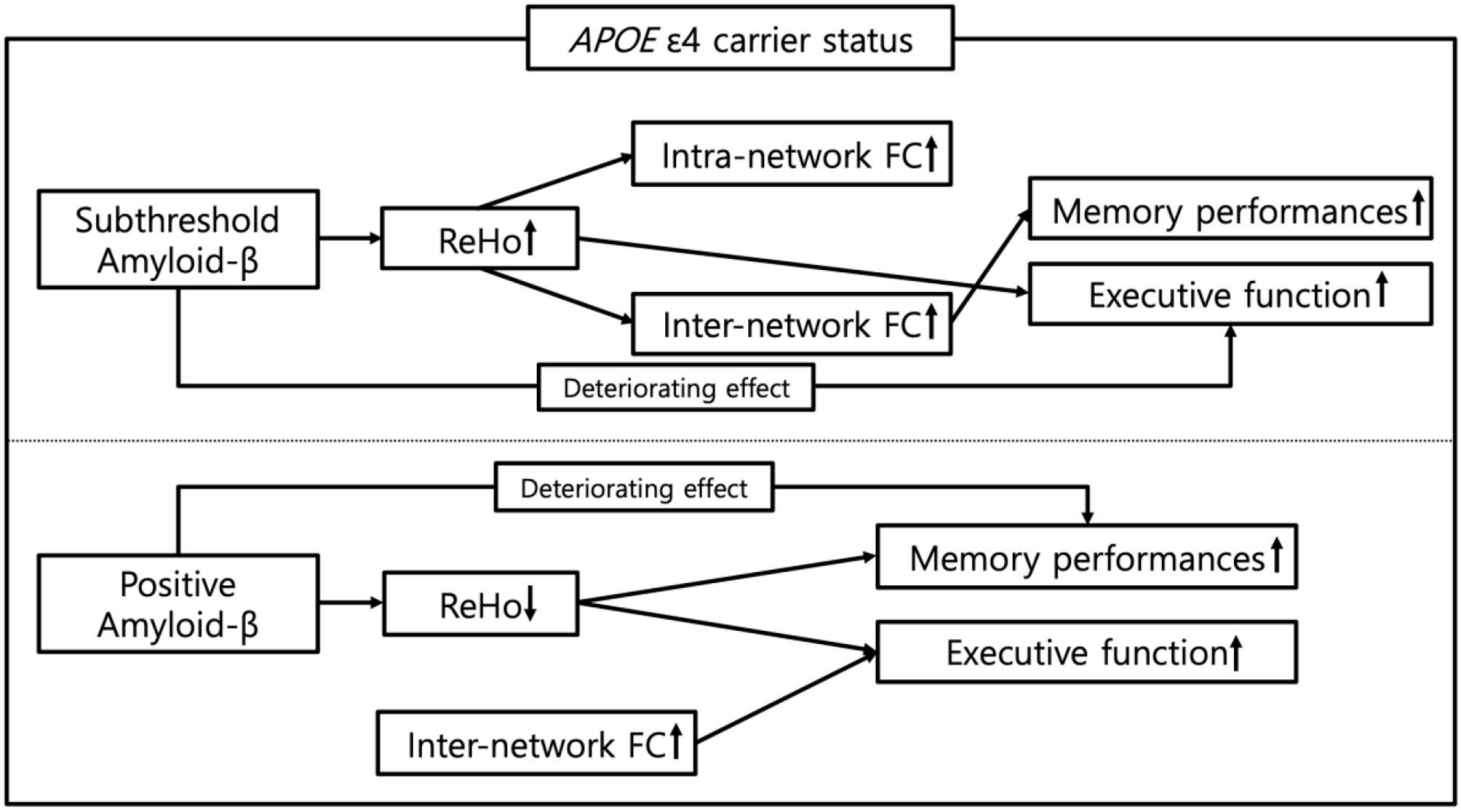
Schematic representation of the impact of *APOE* ε4 carrier status on the pathway from subthreshold and positive Aβ deposition to cognitive functions in cognitively normal older adults. ReHo, regional homogeneity; FC, functional connectivity.

The present study was designed to explore the distinctive effect of the *APOE* ε4 carrier status on the associations between subthreshold, positive Aβ deposition, FC, and cognitive performance during the preclinical phase. We have depicted the impact of the *APOE* ε4 carrier status on the pathway from the subthreshold and positive Aβ deposits to cognitive performance *via* the local and remote FC, as illustrated in the schematic in Figure 5. Finally, this new understanding based on the *APOE* ε4 carrier status should help to improve predictions of the impact of the earliest Aβ accumulation on the progression of AD with a compliment to the aforementioned limitations.

## Data Availability Statement

The datasets generated or analyzed during the current study are not publicly available because of the Patient Data Management Protocol of Yeouido Saint Mary's Hospital, but are available from the corresponding author upon reasonable request.

## Ethics Statement

The studies involving human participants were reviewed and approved by Institutional Review Board of the Catholic University of Korea. The patients/participants provided their written informed consent to participate in this study.

## Author Contributions

DK contributed to conceptualization, methodology, data curation, writing the original draft, visualization, formal analysis, and funding acquisition. S-MW contributed to methodology, data curation, and writing, reviewing, and editing the manuscript. YU contributed to software and investigation. N-YK contributed to methodology and data curation. CL contributed to conceptualization and supervision. HL contributed to conceptualization, methodology, supervision, project administration, funding acquisition, and writing, reviewing, and editing the manuscript. All authors contributed to the article and approved the submitted version.

## Funding

This work was supported by the National Research Foundation of Korea grants funded by the Korean government (Ministry of Science and ICT) (Nos. 2019R1A2C2009100 and 2019R1C1C1007608). The funders had no role in the study design, data collection and analysis, decision to publish, or preparation of the manuscript.

## Conflict of Interest

The authors declare that the research was conducted in the absence of any commercial or financial relationships that could be construed as a potential conflict of interest.

## Publisher's Note

All claims expressed in this article are solely those of the authors and do not necessarily represent those of their affiliated organizations, or those of the publisher, the editors and the reviewers. Any product that may be evaluated in this article, or claim that may be made by its manufacturer, is not guaranteed or endorsed by the publisher.
