# Supplementary material for: Impact of APOE ε4 Carrier Status on Associations Between Subthreshold, Positive Amyloid-β Deposition, Brain Function, and Cognitive Performance in Cognitively Normal Older Adults: A Prospective Study
Source: Front Aging Neurosci. 2022 May 23;14:871323. doi: 10.3389/fnagi.2022.871323 (PMC9168227; doi:10.3389/fnagi.2022.871323)
Supplement: Supplementary file 1 [file Data_Sheet_1.pdf]

Supplemental Materials for "Impact of *APOE* ε4 carrier status on associations between subthreshold, positive amyloid-β deposition, brain function, and cognitive performance in cognitively normal older adults: a prospective study" by Kang et al.

- **Supplementary Methods**
  1. Neuropsychological evaluation
  2. *APOE* genotyping
  3. Structural and functional MRI data acquisition
  4. Functional MRI data preprocessing
  5. Calculation of intra- and inter-network functional connectivity
  6. PET scanners and SUVR calculation
  7. Structural imaging analysis using voxel-based morphometry
- **Supplementary Results**
  1. Voxel-based morphometry
- **Supplementary Tables and Figures**
  - 1. Table S1 Location of the brain regions in resting state brain networks
  - 2. Figure S1 Differences (A) in intra-network functional connectivity and (B) inter-network functional connectivity for cognitively normal older adults with sub-threshold Aβ deposition between *APOE* ε4 non-carriers and carriers
  - 3. Figure S2 Differences (A) in intra-network functional connectivity and (B) inter-network functional connectivity for cognitively normal older adults with positive Aβ deposition between *APOE* ε4 non-carriers and carriers
- **Supplementary References**

1 Supplementary Methods

1.1 Neuropsychological evaluation

Cognitive status was assessed using neuropsychological testing at Yeouido St. Mary's Hospital, The Catholic University of Korea. Cognitive functions in all the subjects were assessed using the Korean version of the Consortium to Establish a Registry for Alzheimer's Disease (CERAD-K), which included verbal fluency (VF), the 15-item Boston Naming Test (BNT), MMSE-K, Word List Memory (WLM), Word List Recall (WLR), Word List Recognition (WLRc), Constructional Praxis (CP), and Constructional Recall (CR). The CERAD is a standardized clinical and neuropsychological assessment battery for the evaluation of patients with AD. The results were reviewed by a neuropsychologist to determine whether there was evidence of cognitive impairment.

The VF score is the number of animal names that the subject could name in one minute. The BNT scores ranged from 0 to 15 points. The MMSE-K score ranged from 0 to 30 points. The WLM scores ranged from 0 to 30 points. The WLR scores ranged from 0 to 10 points. The WLR scores ranged from 0 to 10 points. The WLRc scores ranged from 0 to 10 points. The CP scores ranged from 0 to 11 points. The CR scores ranged from 0 to 11 points.

To evaluate executive function, we performed the Stroop task, which requires pre-directed reactions while suppressing the dominant response, such as letter reading and color reading conditions. In the current study, the Korean Color Word Stroop Test (K-CWST) was used. The subjects were requested to read the color of letters when letters were written in red, blue, yellow, and black colors within a limited time.(Byeon et al., 2017) Additionally, participants carried out trail-making test B, which alternatively measures the time required to connect letters and numbers in the sequence. Finally, the VF test, the subdomain of the CERAD-K battery, counts the number of animals recalled within a minute.

1.2 *APOE* genotyping

DNA was isolated from blood using the QIAmp Blood DNA Maxi Kit (Qiagen, Valencia, CA, USA). Genotypes for two APOE SNPs, rs429358 (E*4) and rs7412 (E*2) were determined using TaqMan SNP genotyping assays (Applied Biosystems, Foster City, CA, USA).

1.3 Structural and functional MRI data acquisition

Imaging data were collected by the Department of Radiology of Yeouido Saint Mary's Hospital at the Catholic University of Korea using a 3T Siemens Skyra MRI machine and a 32-channel Siemens head coil (Siemens Medical Solutions, Erlangen, Germany). The parameters used for the T1-weighted volumetric magnetization-prepared rapid gradient echo scan sequences were TE = 2.6 ms, TR = 1,940 ms, inversion time = 979 ms, FOV = 230 mm, matrix = 256×256, and voxel size = 1.0×1.0×1.0 mm^3. Resting-state fMR images were collected using a T2* weighting gradient echo sequence with TR = 2,000 ms, TE = 30 ms, matrix = 128 × 128 × 29, and voxel size = 1 × 1 × 2 mm^3. We acquired 150 volumes in 5 minutes, with the instruction, "keep your eyes closed and think of nothing in particular."

1.4 Functional MRI data preprocessing

We used the data processing assistant for resting-state fMRI (DPARSF, GNU GENERAL PUBLIC LICENSE, Beijing, China), which is based on Statistical Parametric Mapping (SPM, http://www.fil.ion.ucl.ac.uk/spm, Wellcome Centre for Human Neuroimaging, London, England). Slice timing and realignment for motion corrections were performed on the images. Subjects with excessive head motion (cumulative translation or rotation > 2 mm or 2°) were excluded. To prevent group-related differences caused by microscopic head motion, framewise displacement (FD) was compared between the groups. The mean FD scores did not differ between *APOE* ε4 carriers and non-carriers in each CN sub-Aβ or CN Aβ + group ($P > .05$, 2-sample t test). For spatial registration, T1-weighted images were co-registered to the mean rsfMRI image based on rigid-body transformation. For spatial normalization, the International Consortium for Brain Mapping template was applied (resampling voxel size = 3 × 3 × 3 mm) and fitted to the "East Asian brain" Subsequently, the functional images were spatially smoothed with a 6 mm full width at half maximum Gaussian kernel. For local regional homogeneity (FC), spatial smoothing was performed after a map for local FC was obtained to avoid increasing the regional similarity.(Yan and Zang, 2010) We further processed our functional data to fit them to local and remote FC analyses using DPARSF. Linear trends were removed from the functional images, and data were filtered using a temporal band-pass of 0.01–0.08 Hz, to reduce low-frequency drift as well as physiological high-frequency respiratory and cardiac noise. Several nuisance covariates were regressed out, including six head motion parameters and signals from the white matter and cerebrospinal fluid.

1.5 Calculation of intra- and inter-network functional connectivity

1.5.1 Intra-network functional connectivity(Wang et al., 2015)

$$Z_X = \frac{1}{\frac{n_X(n_X-1)}{2}} \sum_{ij=1:n_X} |z_{i,j}|$$

where n_X is the number of ROIs within a specific network X.

1.5.2 Inter-network functional connectivity(Wang et al., 2015)

$$Z_{X,Y} = \frac{1}{n_X n_Y} \sum_{i \in X, j \in Y} |z_{i,j}|$$

where X and Y denote the network of the three resting-state networks.

1.6 PET scanners and SUVR calculation

1.6.1 PET scanners

Each scanner was commissioned by scanning a NEMA phantom and adjusting the reconstruction parameters to obtain a spatial resolution of ~6.5 mm. This optimization was performed in advance before the scanning of patients, and the images received by GE were not subjected to any further post-processing with regard to spatial resolution.

1.6.2 SUVR calculation

FMM was manufactured, and FMM-PET data were collected and analyzed as described previously (Thurfjell et al., 2014). Static PET scans were acquired from 90 to 110 min after 185 MBq of FMM injection. MRI for each participant was used to co-register and define the ROIs and correct partial volume effects that arose from expansion of the cerebrospinal spaces accompanying cerebral atrophy using a geometric transfer matrix. The semi-quantification of FMM uptake on PET/CT scan was performed by obtaining the standardized uptake value ratios (SUVRs). The volumes of interest (VOIs) were restricted to gray matter, covering the frontal, superior parietal, lateral temporal, anterior, and posterior cingulate cortex/precuneus regions. These VOIs were also considered in a previous study (Thurfjell et al., 2014). The reference region for SUVR calculations was pons. The mean uptake counts of each VOIs and reference region were measured on the preprocessed image. A regional SUVR was calculated as the ratio of each cortical regional mean count to the pons mean count (SUVRPONS). The global cortical average (composite SUVR) was calculated by

averaging regional cortical SUVRs weighted for size. We used a cut-off of 0.62 for “positive” versus ‘sub-threshold’ neocortical SUVR, consistent with the cut-off values used in a previous FMM PET study (Thurfjell et al., 2014). PET scans classified with sub-threshold Aβ accumulation also exhibited normal visual reading.

1.7 Structural imaging analysis using voxel-based morphometry (VBM)

Image preprocessing and analysis were performed using SPM12 (Wellcome Trust Centre for Neuroimaging, London, UK; http://www.fil.ion.ucl.ac.uk/spm) using the CAT12 toolbox (http://www.neuro.uni-jena.de/cat/). We utilized the optimized VBM process(Good et al., 2001) which included 1) segmentation and extraction of the brain in native space, 2) normalization of the images to a Montreal Neurological Institute (MNI) standard space, 3) segmentation and extraction of the normalized brain (extraction was repeated to ensure that no non-brain tissues remained); 4) modulation of the normalized images to correct for tissue volume differences due to the normalization procedure, and 5) sample homogeneity was checked to identify any outliers in the study population. The gray matter probability values were smoothed using an 8-mm full-width half-maximum isotropic Gaussian kernel. The smoothed gray matter images were analyzed using an analysis of covariance model using SPM12. Age, sex, years of education, and total intracranial volume (TIV) were included as covariates. The statistical significance threshold was set at a *P*-value of less than .05, to resolve the problem of multiple comparisons.

2 Supplementary Results

2.1 Voxel based morphometry (VBM)

The VBM analysis revealed no significant differences between the *APOE* ε4 carriers and non-carriers in each CN sub-Aβ and Aβ+ group, indicating that atrophy did not make a marked contribution to the present findings in this study.

3 Supplementary Tables and Figures

Table S1 Locations of brain regions in resting state brain networks(Brier et al., 2012)

ROI	MNI coordinates
Posterior cingulate cortex[a]	0, -51, 29
Medial prefrontal cortex[a]	0, 61, 22
Left lateral parietal[a]	-48, -66, 34
Right lateral parietal[a]	53, -61, 35
Left inferior temporal[a]	-65, -22, -9
Right inferior temporal[a]	61, -21, -12
Medial thalamus[a]	0, -9, 7
Left posterior cerebellum[a]	-28, -82, -32
Right posterior cerebellum[a]	26, -89, -34
Dorsal mPFC[b]	1, 30, 44
Left anterior PFC[b]	-45, 50, -5
Right anterior PFC[b]	46, 51, -7
Left superior parietal[b]	-51, -50, 49
Right superior parietal[b]	53, -49, 47
Right anterior cingulate cortex[c]	12, 32, 30
Left anterior cingulate cortex[c]	-13, 34, 16
Right ventral anterior cingulate cortex[c]	10, 34, -6
Left putamen[c]	-19, 3, 9

Right putamen[c]	25, 18, 8
Left insula[c]	-42, 6, 4
Right insula[c]	43, 7, 2

[a]DMN, default-mode network; [b]CEN, central-executive network; [c]SAL, salience network.

Figure S1 Differences (A) in intra-network functional connectivity and (B) inter-network functional connectivity in cognitively normal older adults with subthreshold Aβ deposition between *APOE* ε4 non-carrier and carrier

(A)

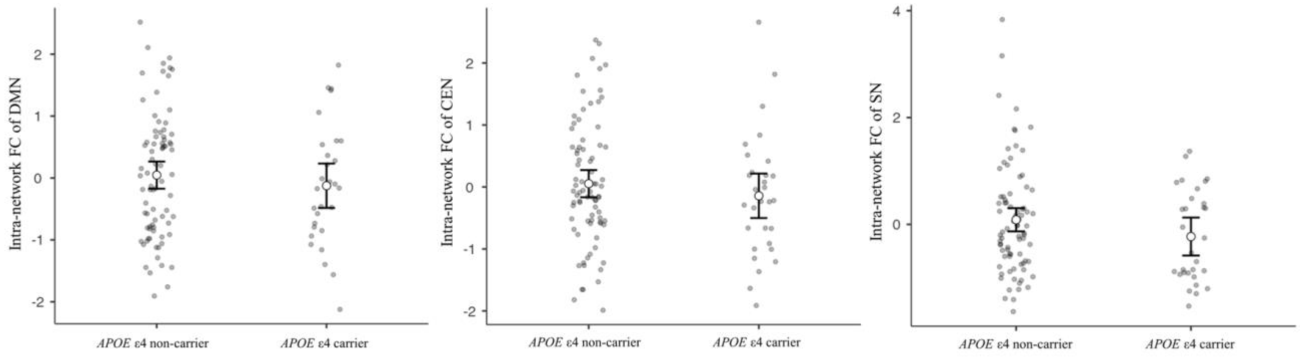

(B)

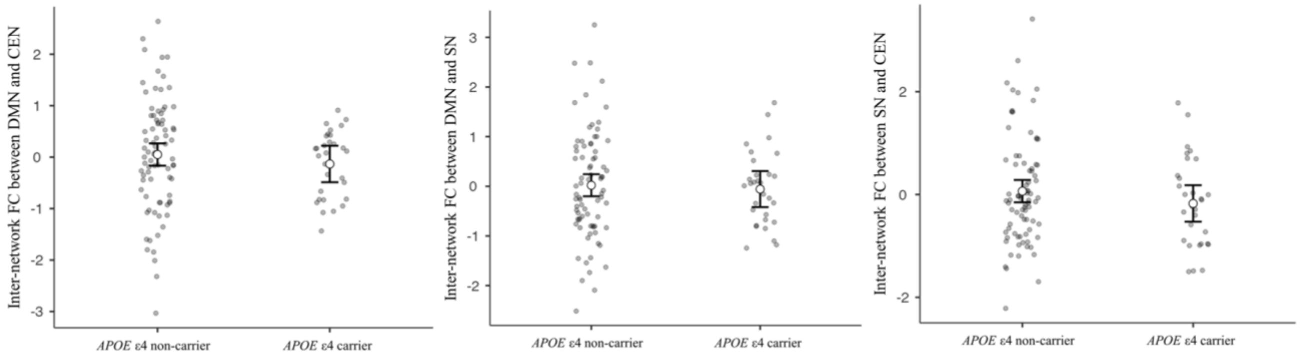

ANCOVA, adjusting for age, sex, and years of education. Each variable was z-transformed using the mean and standard deviation. There are no significant differences in each variable between the groups. FC, functional connectivity; DMN, default-mode network; CEN, central-executive network; SN, salience network.

Figure S2 Differences (A) in intra-network functional connectivity and (B) inter-network functional connectivity in cognitively normal older adults with positive Aβ deposition between *APOE* ε4 non-carrier and carrier

(A)

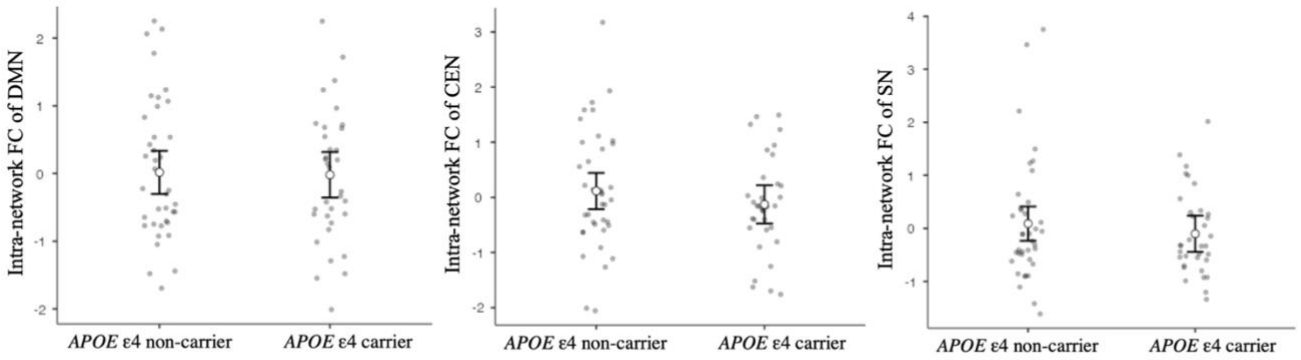

(B)

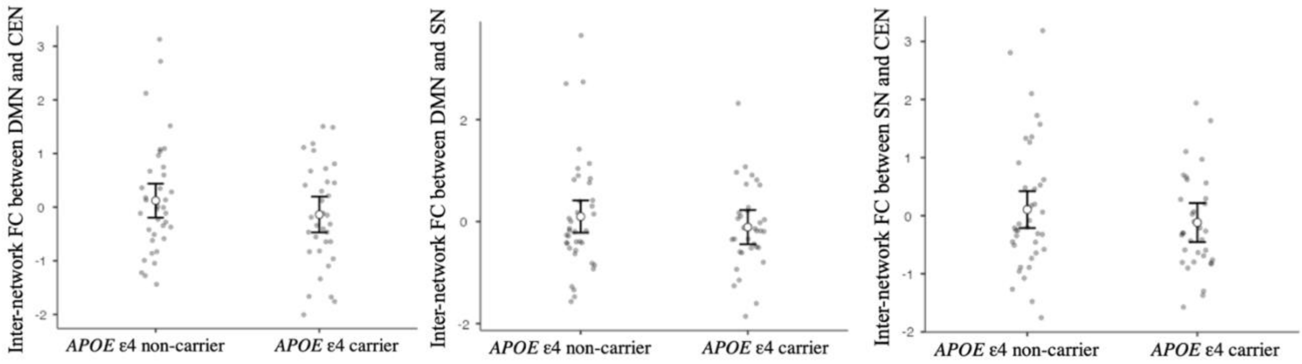

ANCOVA, adjusting for age, sex, and years of education. Each variable was z-transformed using the mean and standard deviation. There are no significant differences in each variable between the groups. FC, functional connectivity; DMN, default-mode network; CEN, central-executive network; SN, salience network.

4 Supplementary References

Brier, M.R., Thomas, J.B., Snyder, A.Z., Benzinger, T.L., Zhang, D., Raichle, M.E., Holtzman, D.M., Morris, J.C., and Ances, B.M. (2012). Loss of intranetwork and internetwork resting state functional connections with Alzheimer's disease progression. *J Neurosci* 32**,** 8890-8899.

Byeon, H., Jin, H., and Cho, S. (2017). Development of Parkinson's disease dementia prediction model based on verbal memory, visuospatial memory, and executive function. *Journal of Medical Imaging and Health Informatics* 7**,** 1517-1521.

Good, C.D., Johnsrude, I.S., Ashburner, J., Henson, R.N., Friston, K.J., and Frackowiak, R.S. (2001). A voxel-based morphometric study of ageing in 465 normal adult human brains. *Neuroimage* 14**,** 21-36.

Thurfjell, L., Lilja, J., Lundqvist, R., Buckley, C., Smith, A., Vandenberghe, R., and Sherwin, P. (2014). Automated quantification of 18F-flutemetamol PET activity for categorizing scans as negative or positive for brain amyloid: concordance with visual image reads. *J Nucl Med* 55**,** 1623-1628.

Wang, P., Zhou, B., Yao, H., Zhan, Y., Zhang, Z., Cui, Y., Xu, K., Ma, J., Wang, L., An, N., Zhang, X., Liu, Y., and Jiang, T. (2015). Aberrant intra- and inter-network connectivity architectures in Alzheimer's disease and mild cognitive impairment. *Sci Rep* 5**,** 14824.

Yan, C., and Zang, Y. (2010). DPARSF: a MATLAB toolbox for" pipeline" data analysis of resting-state fMRI. *Frontiers in systems neuroscience* 4**,** 13.
